# Dual-defense functionality of temporin-1LS from dark-spotted frogs (*Pelophylax nigromaculatus*): antimicrobial activity and immunomodulatory effects via macrophage activation

**DOI:** 10.3389/fimmu.2026.1829966

**Published:** 2026-04-10

**Authors:** Li Ma, Shengmin Wu, Hui Hu, Yonghui Zhang, Yang Gong, Wei Sun, Ling Guo, Zhihua Lin, Jie Chen

**Affiliations:** 1 College of Agriculture and Biotechnology, Lishui University, Lishui, China; 2 Nanjing Institute of Environmental Sciences, Ministry of Ecology and Environment, Nanjing, China; 3 Industrial College of Traditional Chinese Medicine and Health, Lishui University, Lishui, China

**Keywords:** antimicrobial peptide, dual functionality, immunomodulation, macrophage, pelophylax nigromaculatus, temporin-1LS

## Abstract

**Introduction:**

The escalating crisis of antibiotic resistance highlights the urgent need for novel antimicrobial agents. This study aims to identify and characterize temporin-1LS, a temporin-family antimicrobial peptide from the dark-spotted frog (*Pelophylax nigromaculatus*), and to investigate its biological functions, including antimicrobial and immunomodulatory activities.

**Methods:**

Structural and phylogenetic analyses were conducted to determine the peptide’s conformation and evolutionary relationship. Expression levels of temporin-1LS in various tissues were assessed under basal and pathogen-challenged conditions using *Aeromonas hydrophila*. The antimicrobial activity of synthetic temporin-1LS was evaluated by determining minimum inhibitory concentrations (MICs) against bacterial strains. Immunomodulatory functions, including macrophage chemotaxis, phagocytosis, and respiratory burst, were examined *in vitro*.

**Result:**

Temporin-1LS possesses a conserved amphipathic α-helical structure and shows high phylogenetic homology with temporin-1GY. It was constitutively highly expressed in frog skin and significantly upregulated in the gut (47.4-fold) and liver (24.3-fold) following *A. hydrophila* challenge. The synthetic peptide exhibited potent and selective antibacterial activity against *Staphylococcus warneri* and *Vibrio alginolyticus* (MIC = 3.125 µg/mL). Temporin-1LS significantly increased bacterial LDH release at higher concentrations (50–100 μg/mL) relative to the BSA control, suggesting possible membrane damage under these conditions. Although it did not show measurable *in vitro* antibacterial activity against *A. hydrophila*, temporin-1LS administration significantly improved survival in a frog infection model. Furthermore, the peptide demonstrated strong immunomodulatory effects, stimulating macrophage chemotaxis (2.4-fold), enhancing phagocytosis (4.4-fold), and amplifying respiratory burst (1.6-fold) in a dose-dependent manner.

**Discussion and conclusions:**

Temporin-1LS employs a dual-defense mechanism, combining direct antibacterial activity against susceptible pathogens with strategic enhancement of key macrophage functions to resolve infection. These findings position temporin-1LS as a promising scaffold for developing novel immunomodulatory anti-infectives.

## Introduction

Against the backdrop of the increasingly severe global challenge of antibiotic resistance, the development of novel antimicrobial agents with distinct mechanisms of action has become an urgent priority (1). Amphibian skin secretions, particularly those from anuran species, represent a rich repository of antimicrobial peptides (AMPs) due to their long-term evolution in microbe-rich environments, making them a crucial source for discovering natural antimicrobial molecules (2, 3). Among these, the temporin family, a class of short antimicrobial peptides (10–14 amino acid residues), not only exhibits broad-spectrum antimicrobial activity but also demonstrates immunomodulatory functions, positioning them as ideal candidate molecules for developing dual-function agents with both antimicrobial and immunoregulatory properties (4–6).

Since their initial isolation from the common frog (*Rana temporaria*), temporins have been shown to primarily act by targeting microbial cell membranes, a mechanism that reduces the likelihood of bacterial resistance development while exhibiting low toxicity toward mammalian cells (7). In recent years, temporin homologs identified from species such as the Sahara frog (*Pelophylax saharica*) (8), the Yunnan odorous frog (*Odorrana grahami*) (9) and the broad-folded frog (*Hylarana latouchii*) (10) have further validated their conserved structural motifs and expanded their pharmacological profiles. For instance, temporin-SHa from the Sahara frog is a well-characterized representative of temporin. It has been reported to exhibit broad anti-infective activity, and SHa-derived analogs have also been investigated for expanded bioactivities in recent studies. In particular, temporin-SHa and its analogs demonstrated strong antibacterial efficacy against *Helicobacter pylori*, including resistant clinical strains, highlighting the translational relevance of SH-family temporins (8). In addition, SHa-related peptides have been investigated in antifungal/anti-biofilm contexts, further supporting the functional diversity within the temporin family (11, 12). Meanwhile, temporin A from the common frog shows synergistic effects with conventional antibiotics (13). Despite these findings, the functional diversity within the temporin family, particularly their immunomodulatory capabilities in vertebrate models, remains poorly understood (14).

Recent advances in host defense peptide research have highlighted the intricate relationship between their direct microbicidal activity and immunomodulatory functions (15). Beyond direct microbial killing, AMPs such as cathelicidins and β-defensins can activate macrophages via TLR4/NF-κB and MAPK signaling pathways, enhancing phagocytosis, promoting cytokine production (e.g., TNF-α, IL-6), and improving antigen presentation capacity (16–19). This “dual-defense” characteristic makes AMPs promising candidates for the treatment of infectious diseases and immune dysregulation-related conditions (20). However, evidence regarding the immunostimulatory effects of temporins remains limited. Existing studies indicate that temporin A can induce monocyte chemotaxis through formyl peptide receptor activation (21).

The dark-spotted frog (*Pelophylax nigromaculatus*), a widely distributed amphibian species in East Asia, represents a potential reservoir of AMP resources. Although several AMPs have been identified in this species (22), systematic research on its temporin family remains scarce. This study aims to fill this gap by identifying and characterizing a new temporin homolog, temporin-1LS, from the dark-spotted frog. Combining structural and functional analyses, we systematically evaluated the antimicrobial activity of this mature peptide and its immunomodulatory potential on macrophages. This work not only expands the understanding of the biological functions of temporins within amphibian defense systems but also provides a scientific foundation for developing targeted anti-infective therapeutics with dual antimicrobial and immunomodulatory functions.

## Methods

### Experimental animals

Dark-spotted frogs of 100 ± 10 g were obtained from an aquaculture facility in Lishui, China. The animals were housed in temperature - controlled freshwater tanks (23–25 °C) at a density of 20 frogs per tank, under a natural light-dark cycle. The water *p*H was maintained at approximately 6.5–7.0, and the dissolved oxygen was kept at about 7 mg/L. The animals were fed a commercial, pelleted diet twice daily. Prior to experimentation, all specimens underwent a two-week acclimation period under controlled laboratory conditions. All experimental animal procedures were conducted in full compliance with the Lishui University Guidelines for the Care and Use of Laboratory Animals and were reviewed and approved by the relevant institutional authority (Approval No. LSU-AREC-202412012).

### Molecular characterization of temporin-1LS cDNA

The cDNA sequence encoding temporin-1LS was identified using skin transcriptome analysis. ProtParam facilitated computation of the peptide’s molecular mass and *p*I, whereas SignalP 5.0 was used to predict signal peptide cleavage sites. Structural modeling of the peptide was performed using AlphaFold2 (23). HeliQuest generated helical wheel diagrams to visualize amphipathic properties, and ClustalW executed multiple sequence alignments. Phylogenetic reconstruction was conducted with MEGA X, using neighbor-joining methodology.

### Constitutive and induced expression of the temporin-1LS gene

To investigate constitutive expression patterns, eight organ specimens (liver, spleen, kidney, lung, gut, skin, heart, and muscle) were harvested from four healthy dark-spotted frogs. For pathogenic challenge studies, experimental animals (n = 8) received intraperitoneal injections of *A. hydrophila* (1.0 × 10^4^ colony forming units, CFU) following established protocols (24), with control groups (n = 4) administered equivalent volumes of sterile saline. Selected tissues (liver, spleen, kidney, skin, and gut) were collected from four infected frogs at 12 h post-infection and from the remaining four infected frogs at 24 h post-infection. Because tissue sampling was terminal, different animals were used at the two time points. For each tissue, four individual frogs constituted four biological replicates for expression analysis. The collected tissues were snap-frozen in liquid nitrogen for subsequent cryopreservation at -80 °C.

### qPCR

For qPCR analysis, total RNA was prepared with TRIzol (Beyotime, Shanghai, China). Complementary DNA was generated by reverse transcription using the PrimeScript RT reagent kit (TaKaRa, Dalian, China), which includes a gDNA Eraser module for genomic DNA removal. Quantitative PCR analysis was conducted on a CFX96 system (Bio-Rad, Hercules, USA) with BeyoFast™ SYBR Green Mix (Beyotime), detecting cycle thresholds for both *temporin-1LS* and the endogenous control, *PnGAPDH* (**Table 1**). Gene expression quantification was achieved using the 2^-ΔΔCt^ method (25).

**Table 1:** Oligonucleotide primers.

Gene	Primers	Sequence (5′-3′)
*temporin-1LS*	temporin-1LS-t (+)	CTCCTTTTCTTCCTTGGAACCA
	temporin-1LS-t (-)	TCCCCAACAAACTAGAGAGCA
*GAPDH*	GAPDH-t (+)	ATCCCTGCTCTGAACGGAAA
	GAPDH-t (-)	ATTCCCTTCAGTGGTCCCTG

### Antibacterial assay

The bioactive form of temporin-1LS (primary sequence: VIPIVSGLLSSLL-NH_2_) was produced using solid-phase synthesis (SynPeptide Co., Ltd., China) with purity exceeding 95%. Antibacterial efficacy assessment involved seven reference strains: gram-positive *Staphylococcus warneri* and *Staphylococcus aureus* and gram-negative pathogens including *Shigella flexneri*, *Vibrio alginolyticus*, *Escherichia coli*, *A. hydrophila*, and *Proteus mirabilis*. Temporin-1LS was dissolved in sterile water (1 mg/mL stock) with vortexing until fully solubilized, and no visible precipitation was observed before dilution for MIC testing. Quantitative antibacterial evaluation was performed using a broth microdilution assay, as previously described (26). Sterile 96-well microplates were loaded with a 1:2 stepwise dilution gradient starting at 1 mg/mL. Reactions were set up by adding 10 μL of each dilution to 90 μL of exponentially growing bacteria (1×10^5^ CFU/mL) per well. Chloramphenicol (Sigma) was included as a positive control in the broth microdilution assay and was serially diluted in parallel with the peptide using the same two-fold dilution procedure. PBS was used as the vehicle control. Each concentration was tested in triplicate, and the assay was repeated independently three times. Following 24 h incubation at species-specific temperatures, minimum inhibitory concentration (MIC) values were determined by spectrophotometric quantification (OD_600_) using a microplate reader. The antibacterial activity was determined by the MIC, defined here as the lowest peptide concentration causing an OD_600_ value less than 50% of the growth control after incubation (27, 28).

### Lactate dehydrogenase release assay

To evaluate bacterial membrane damage, LDH release was quantified using a commercial LDH assay kit (Beyotime, Shanghai, China). Briefly, S. warneri cells (1 × 10^9^ CFU) were harvested and resuspended in PBS. The suspension was then treated with temporin-1LS at final concentrations of 25, 50, or 100 µg/mL and incubated at 37 °C for 2 h, with BSA (100 µg/mL) as the protein control. After treatment, 60 µL of LDH working reagent was added to each well, and the plates were kept at room temperature for 30 min. The absorbance was subsequently recorded at 490 nm. LDH release was normalized to the 100 µg/mL BSA-treated control (set as 1.0).

### Hemolysis assay

Following a previously reported temporin hemolysis protocol (29), hemolytic activity of temporin-1LS was tested using defibrinated horse erythrocytes (TCS Biosciences, Buckingham, UK). Erythrocytes were washed with PBS (pH 7.4) and resuspended to 2% (v/v). Temporin-1LS was dissolved in sterile water (1 mg/mL stock) and diluted in PBS to final concentrations of 100, 50, 25, 12.5, 6.25, and 3.125 μg/mL. Peptide solutions were incubated with erythrocytes at 37 °C for 1 h. PBS and 1% Triton X-100 (final concentration) were used as the negative and positive controls, respectively. After centrifugation, hemoglobin release in the supernatant was measured at 540 nm. Hemolysis (%) was calculated by normalizing sample absorbance to the PBS (0%) and Triton X-100 (100%) controls. HC_50_ was determined from the concentration–response curve, and assays were performed in triplicate in three independent experiments.

### Frog survival assays

Frogs (n = 48) were randomly distributed into three groups of 16 animals. Each individual was experimentally infected with *A. hydrophila* by administering 100 μL of inoculum containing 1.0 × 10^4^ CFU. Therapeutic intervention was initiated 30 min after challenge: two groups were treated intraperitoneally with temporin-1LS at 0.1 μg/g or 1.0 μg/g, respectively. For the control condition, frogs received intraperitoneal BSA at 1.0 μg/g as the protein control. Survival was tracked over a 7-day period, and animal condition was inspected at 12 h intervals throughout. Frogs that survived to the end of the study were euthanized.

### Cell preparation

Splenic macrophages from dark-spotted frogs were isolated with minor modifications to a previously described method (30). Briefly, an undiluted Percoll stock solution was prepared by mixing 1.5 M NaCl with 100% Percoll at a 9:1 (v/v) ratio. Discontinuous gradients were then prepared using 0.15 M NaCl: 40% Percoll (6.14 mL 0.15 M NaCl + 3.86 mL 100% Percoll; buoyant density ≈ 1.056 g/mL) and 30% Percoll (7.14 mL 0.15 M NaCl + 2.86 mL 100% Percoll; buoyant density ≈ 1.043 g/mL). Frogs were anesthetized by immersion in 0.6 g/L MS-222 buffered with 0.6 g/L sodium bicarbonate, disinfected with 75% ethanol, and euthanized while unconscious. Spleens were aseptically excised, rinsed three times with sterile PBS, and washed twice with serum-free medium. The tissue was finely minced, passed through a 70 μm cell strainer, and centrifuged at 1000 rpm for 5 min. The pellet was resuspended and layered onto the 40%/30% Percoll gradients. After density-gradient centrifugation, the cell fraction located above the 30% Percoll layer was collected. Cells were resuspended in M199 medium supplemented with penicillin (1000 U/mL), streptomycin (1000 μg/mL), and 20% fetal bovine serum (FBS), and cultured at 25 °C under 2% CO_2_. Enriched macrophages were seeded at 5 × 10^6^ cells per plate and incubated for 12 h to allow adherence, after which non-adherent cells were removed. Cell viability and concentration were determined using the trypan blue exclusion assay prior to downstream experiments.

### CCK-8 assay

Cell viability was determined using a CCK-8 assay kit (Elabscience Biotechnology, Wuhan, China). Macrophages (5 × 10³ cells/well) were seeded into 96-well plates and exposed to temporin-1LS at 0.1, 1.0, or 10.0 μg/mL for 24 h; BSA (10.0 μg/mL) was included as the protein control. After treatment, 10 μL of CCK-8 reagent was added to each well, followed by incubation for 4 h. Absorbance was then recorded at 450 nm using a microplate reader.

### Chemotactic assay

To assess the chemotactic potential of temporin-1LS, a chamber-based migration assay was performed (31). The experimental system used 24-well Transwell inserts (Corning; 5 μm pore diameter) within a humidified incubator (25 °C, 2% CO_2_). Macrophages were suspended in serum-free M199 medium at 6 × 10^4^ cells/well in the upper compartment; the lower chamber contained temporin-1LS dissolved in complete M199 at three logarithmic increments (0.1–10.0 μg/mL), while BSA (10.0 μg/mL) dissolved in complete M199 served as the protein control. Following 24-h chemoattraction, polycarbonate membranes were methanol-fixed and stained with Wright–Giemsa stain. Transmigrated cells were quantified using light microscopy (Nikon Eclipse Ti2; 400×) across five randomized fields per insert, with the chemotactic index calculated as the treated migration divided by the basal migration. The experimental design included triplicate wells per concentration group with three biological replicates.

### Phagocytosis assay

To investigate the immunomodulatory effects of temporin-1LS on macrophage function, a fluorescence-activated cell sorting (FACS)-based phagocytosis quantification adapted from established protocols was conducted (32). Macrophages (5×10^5^ cells/well) were primed with temporin-1LS (0.1, 1.0, and 10.0 μg/mL) in complete M199 (20% heat-inactivated FBS) for 12 h under physiological opsonization conditions. Post-treatment, three consecutive ice-cold PBS washes were performed to eliminate residual peptides. Phagocytic capacity was assessed by incubating cells with FITC-conjugated dextran (1 mg/mL) in serum-deprived M199 for 30 min at 25 °C in a 2% CO_2_ atmosphere. Non-internalized fluorophores were quenched with acidic trypan blue solution (0.4% w/v, *p*H 4.4) for 60 s. Cellular fluorescence was quantified using a Beckman CytoFLEX LX (B51530) flow cytometer. Phagocytic index was normalized against the BSA-based vehicle control (0.1% BSA-PBS), which was set as the baseline (100), and expressed as relative MFI fold-change. The experiment included triplicate technical replicates validated in three independent biological replicates.

### Respiratory burst assay

To evaluate the modulatory effects of temporin-1LS on the phagocyte oxidative burst response, a spectrophotometric quantification of superoxide anion (O^2-^) generation was done using a nitroblue tetrazolium chloride (NBT) reduction assay, with methodological adaptations from prior research (33). Macrophages were preincubated with temporin-1LS (0.1, 1.0, and 10.0 μg/mL) in complete M199 medium for 12 h at 25 °C/2% CO_2_. BSA (10.0 μg/mL) in complete M199 medium was used as the protein control. Following phorbol 12-myristate 13-acetate (PMA, 100 ng/mL; Sigma-Aldrich) stimulation (30 min), cells were exposed to 0.1% NBT solution (w/v in HBSS) for 60 min at 24 ± 0.5 °C. The redox reaction was terminated with ice-cold methanol (≥99.9% purity), and intracellular formazan deposits were solubilized in 2 M KOH/DMSO (1:1 v/v; Thermo Fisher). Absorbance measurements at 620 nm were recorded using a microplate reader. All data points represent triplicate technical replicates from three independent biological experiments.

### Statistical analysis

Data are presented as mean ± SEM. For non-survival data, statistical comparisons were performed by one-way ANOVA using SPSS v13.0, and differences were considered significant at *P* < 0.05. Survival data were analyzed using the Kaplan–Meier method, and differences among groups were assessed using the log-rank (Mantel–Cox) test. A *P* value < 0.05 was considered statistically significant.

## Results

### Molecular characterization of temporin-1LS

**Figure 1:**
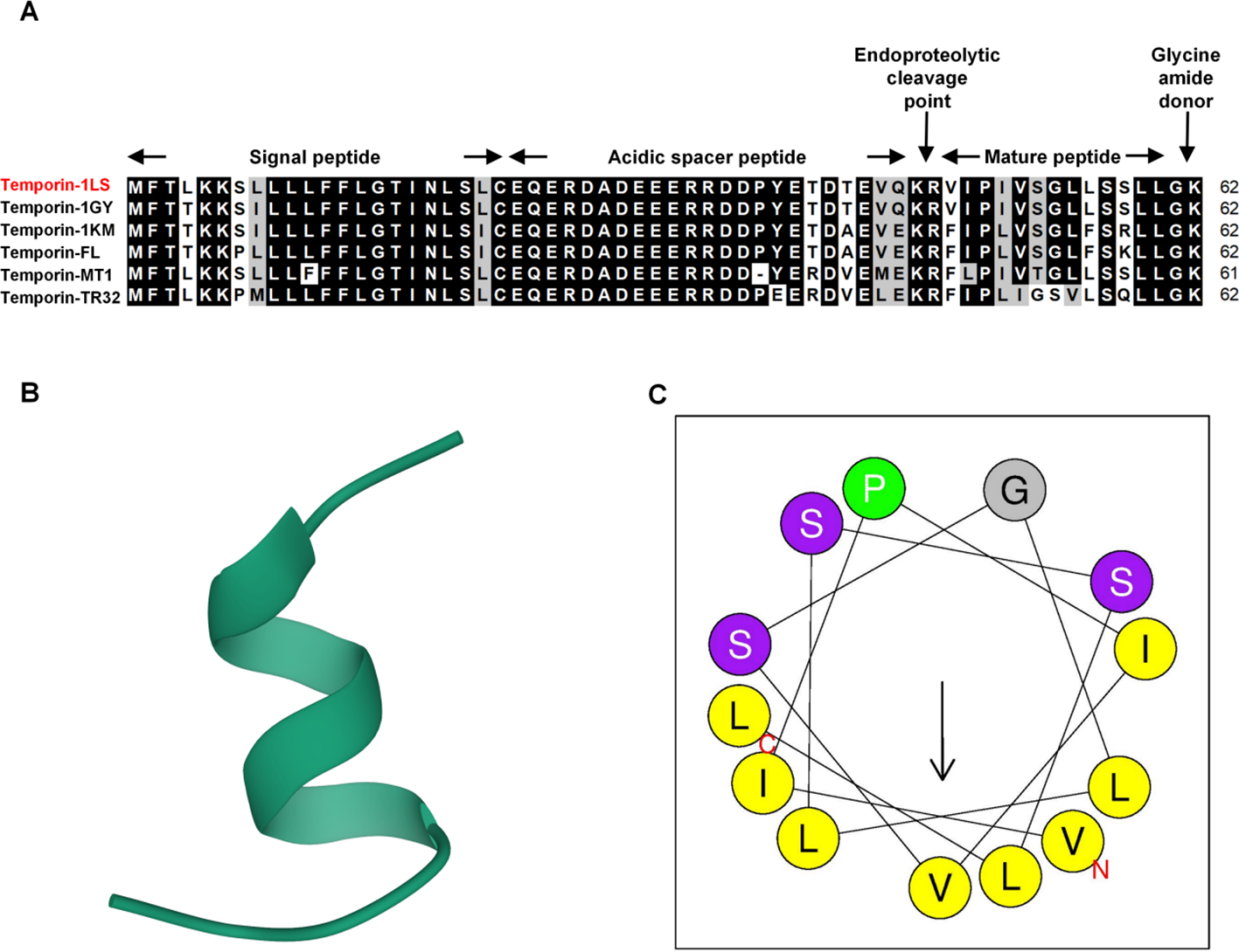
Structural and sequence characterization of temporin-1LS. **(A)** Multiple sequence alignment of temporin-1LS with homologous peptides. Identical and similar residues are highlighted in black and gray, respectively (70% shading threshold). Gaps in the alignment are indicated by hyphens. **(B)** In silico predicted three-dimensional model of temporin-1LS generated using AlphaFold2. **(C)** Sequence-based helical wheel projection generated by HeliQuest, illustrating the predicted amphipathic residue distribution.

The temporin-1LS nucleotide sequence has been submitted to GenBank under accession number PQ826831. Sequence analysis indicated an open reading frame (ORF) spanning 189 nucleotides encoding a 62-amino acid precursor protein. This precursor consisted of three functional domains: a characteristic signal peptide, an intervening spacer acidic peptide, and a bioactive mature peptide. The 13-residue mature peptide contained a C-terminal Gly–Lys motif acting as a proteolytic recognition site for enzymatic amidation. Calculated physicochemical parameters yielded a molecular weight of 1.3 kDa and an isoelectric point (*p*I) of 5.5. Multiple sequence alignment demonstrated strong amino acid sequence conservation among amphibian temporin family members (**Figure 1A**). In silico structural modeling using AlphaFold2 predicted a predominantly α-helical conformation for temporin-1LS (**Figure 1B**). A sequence-based helical wheel projection generated by HeliQuest suggested an amphipathic residue distribution, with hydrophobic residues mainly distributed on one face and polar residues on the opposite face (**Figure 1C**).

**Figure 2:**
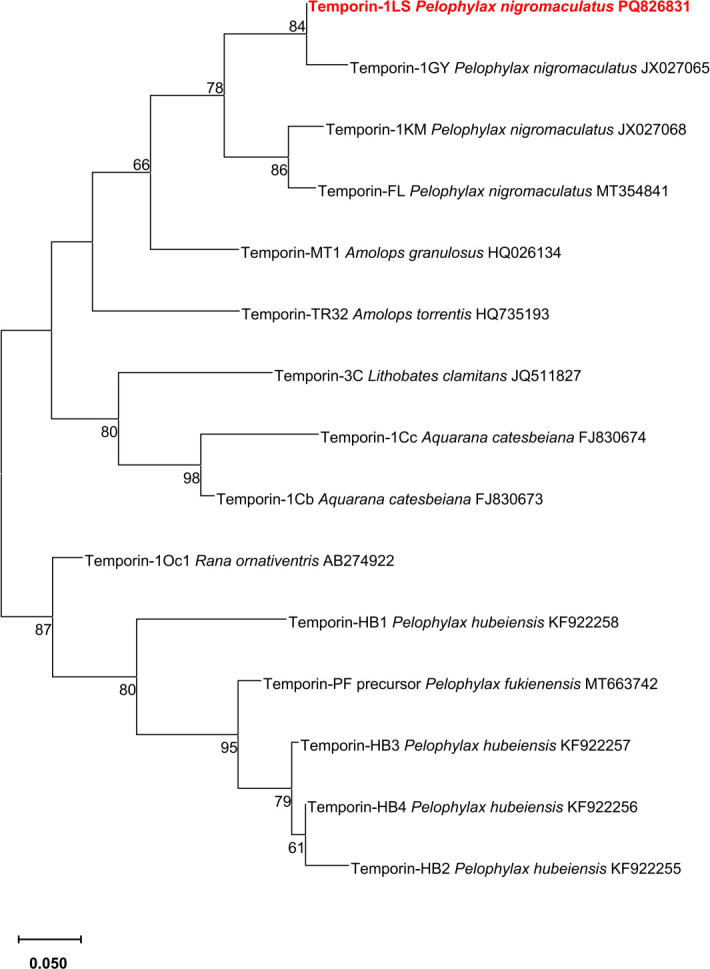
Phylogenetic reconstruction of temporin-1LS amino acid sequences using neighbor-joining method in MEGA X. Bootstrap values (> 60%) from 1000 replicate analyses are shown at branch nodes. The scale bar represents the number of amino acid substitutions per site.

Phylogenetic analysis indicated that temporin-1LS exhibited the highest sequence homology with temporin-1GY (GenBank accession no. JX027065), clustering within the same evolutionary branch as the amphibian AMP from dark-spotted frog (bootstrap value = 84%) (**Figure 2**).

### Constitutive and induced expression profiles of temporin-1LS

**Figure 3:**
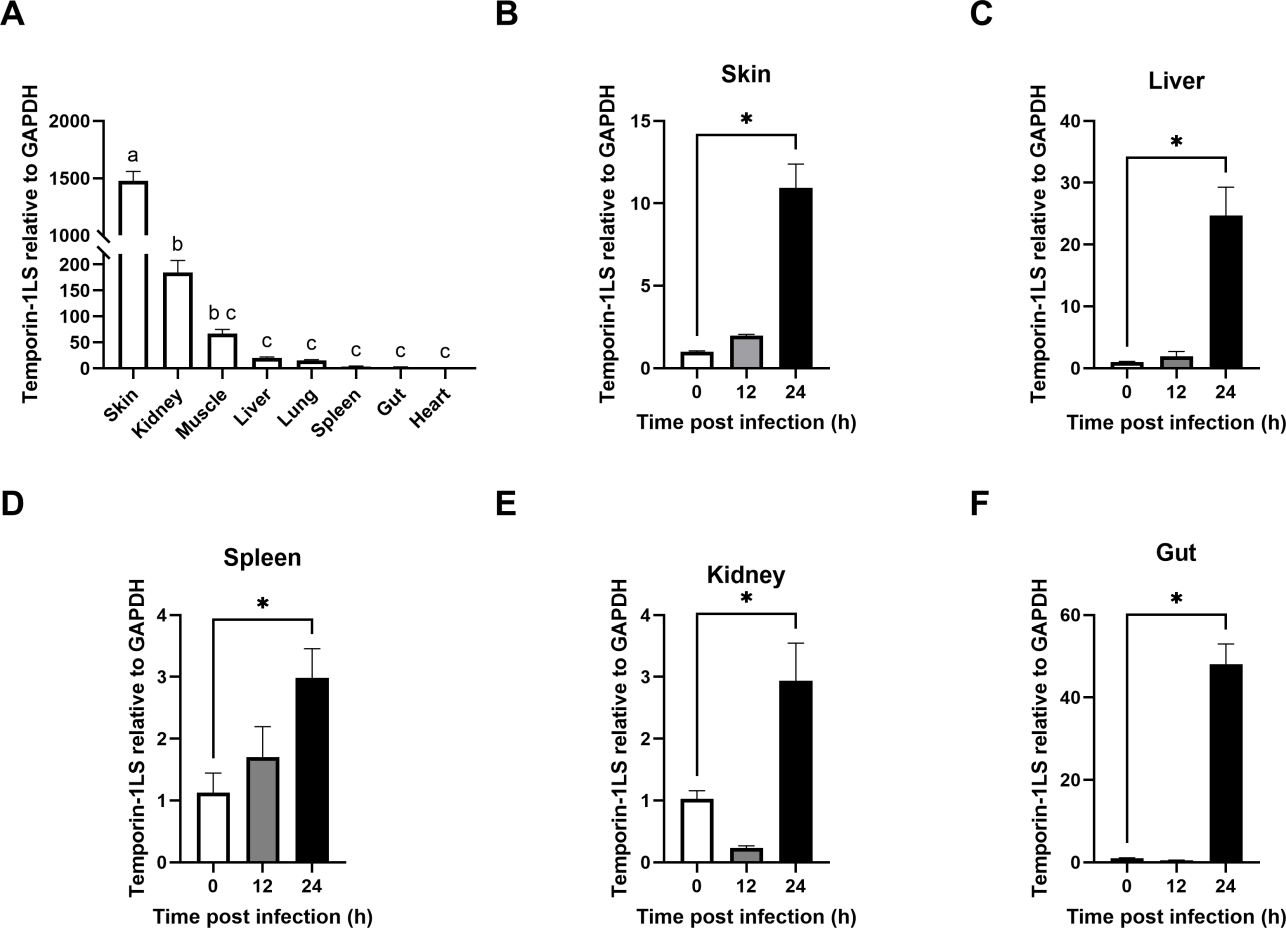
Expression profiling of *temporin-1LS*. **(A)** Constitutive tissue distribution showing differential mRNA abundance (letters denote significant variations at *P* < 0.05 using one-way ANOVA). **(B–F)** Pathogen-responsive expression patterns post-*Aeromonas hydrophila* challenge, relative to endogenous reference (GAPDH). Values represent mean ± SEM (n = 4) with asterisks indicating statistical significance versus control (*P < 0.05, one-way ANOVA).

Basal expression analysis indicated ubiquitous *temporin-1LS* transcription across the examined tissues, with most pronounced accumulation in skin (1,354.8-fold higher than the heart; **Figure 3A**). Challenge with *A. hydrophila* elicited marked induction kinetics, showing tissue-specific amplification patterns: skin (10.9-fold), liver (24.3-fold), spleen (2.6-fold), kidney (2.9-fold), and gut (47.4-fold) (**Figures 3B–F**).

### Antibacterial efficacy of temporin-1LS

The mature temporin-1LS peptide exhibited a selective antibacterial profile against the tested strains (**Table 2**). Potent bacteriostatic activity was observed against *S. warneri* and *V. alginolyticus*, with an MIC of 3.125 μg/mL, which was comparable to that of chloramphenicol. Moderate growth inhibition occurred in *S. aureus* and *S. flexneri* at 12.5 μg/mL, whereas chloramphenicol showed a lower MIC of 3.125 μg/mL against both strains. No measurable inhibition was detected against *E. coli*, *A. hydrophila*, or *P. mirabilis* at the maximum tested concentration (100 μg/mL), while chloramphenicol remained active against these strains.

**Table 2:** Antibacterial activity of synthetic temporin-1LS mature peptide.

Bacteria	Isolate/strain	Temporin-1LS MIC (μg/mL)	Chloramphenicol MIC (μg/mL)
*Staphylococcus warneri*	ATCC49454	3.125	3.125
*Staphylococcus aureus*	ATCC6538	12.5	3.125
*Shigella flexneri*	ATCC12022	12.5	3.125
*Vibrio alginolyticus*	ATCC17749	3.125	3.125
*Escherichia coli*	K12	–	6.25
*Aeromonas hydrophila*	ATCC7966	–	3.125
*Proteus mirabilis*	ATCC25933	–	12.5

– denotes absence of bacteriostatic effects at the maximum tested concentration (100 μg/mL).

### Effects of temporin-1LS on LDH release from *Staphylococcus warneri*

**Figure 4:**
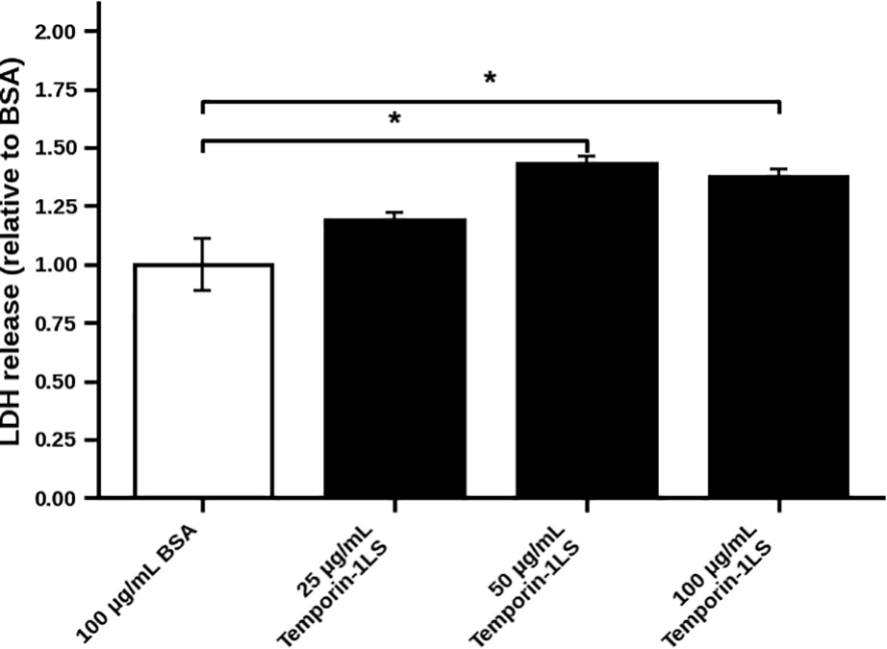
Effect of temporin-1LS on *Staphylococcus warneri* membrane integrity. LDH leakage is presented as fold change, normalized to the BSA-treated negative control (set to 1.0). Values are shown as mean ± SEM (n = 4); *P < 0.05.

LDH release assay was performed to assess *S. warneri* membrane damage induced by temporin-1LS (**Figure 4**). LDH signal was normalized to the 100 μg/mL BSA group (set as 1.0). Temporin-1LS increased LDH release in a dose-dependent manner, reaching 1.19 ± 0.03, 1.43 ± 0.03, and 1.37 ± 0.03 at 25, 50, and 100 μg/mL, respectively, compared with the BSA control (1.00 ± 0.11). These results suggest that temporin-1LS may cause membrane damage in S. warneri at higher concentrations.

### Hemolytic activity of temporin-1LS

**Figure 5:**
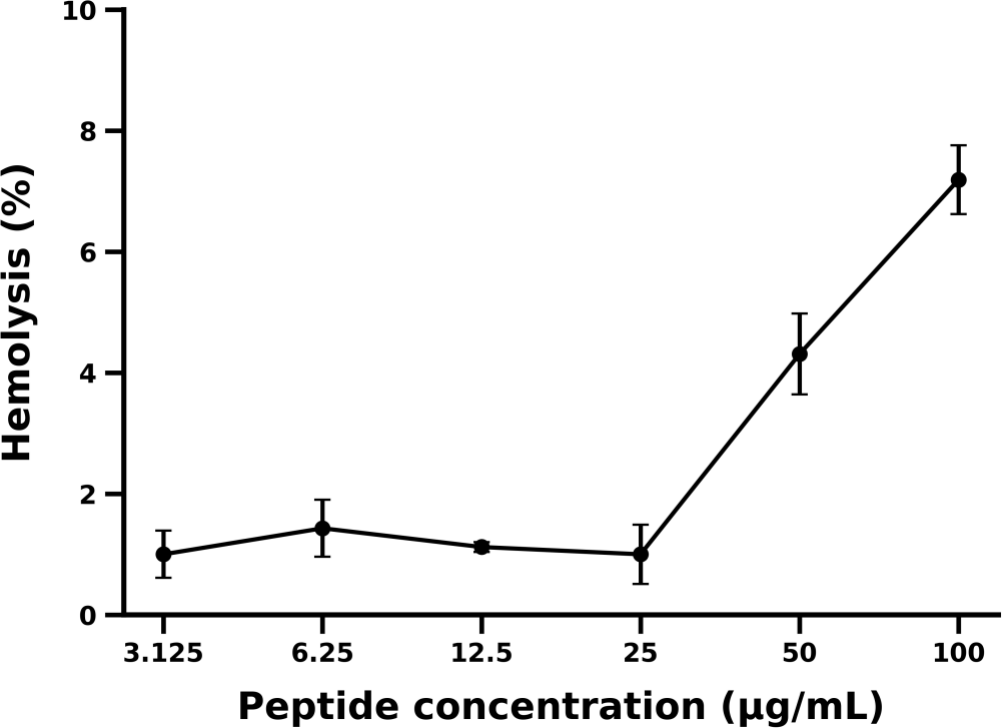
Hemolytic activity of temporin-1LS against horse erythrocytes. Temporin-1LS was incubated with horse erythrocytes at concentrations of 3.125–100 μg/mL for 1 h at 37 °C. Hemolysis was quantified by measuring hemoglobin release at 540 nm and normalized to the PBS (0% hemolysis) and Triton X-100 (100% hemolysis) controls. Data are presented as mean ± SEM (n = 3).

The hemolytic activity of temporin-1LS was evaluated using horse erythrocytes over the concentration range of 3.125–100 μg/mL (**Figure 5**). Temporin-1LS showed limited hemolytic activity under the tested conditions. Hemolysis remained low at all concentrations tested, with mean values of 1.00 ± 0.39% (3.125 μg/mL), 1.43 ± 0.47% (6.25 μg/mL), 1.12 ± 0.08% (12.5 μg/mL), 1.00 ± 0.49% (25 μg/mL), 4.31 ± 0.67% (50 μg/mL), and 7.19 ± 0.57% (100 μg/mL) (**Figure 5**). A slight concentration-dependent increase in hemolysis was observed at higher concentrations (50–100 μg/mL), but hemolysis remained below 10% even at 100 μg/mL. These results indicate that temporin-1LS exhibits low erythrocyte membrane toxicity within the tested concentration range. The HC_50_ was not reached, indicating HC_50_ > 100 μg/mL.

### Effect of the temporin-1LS on frog survival after *A. hydrophila* challenge

**Figure 6:**
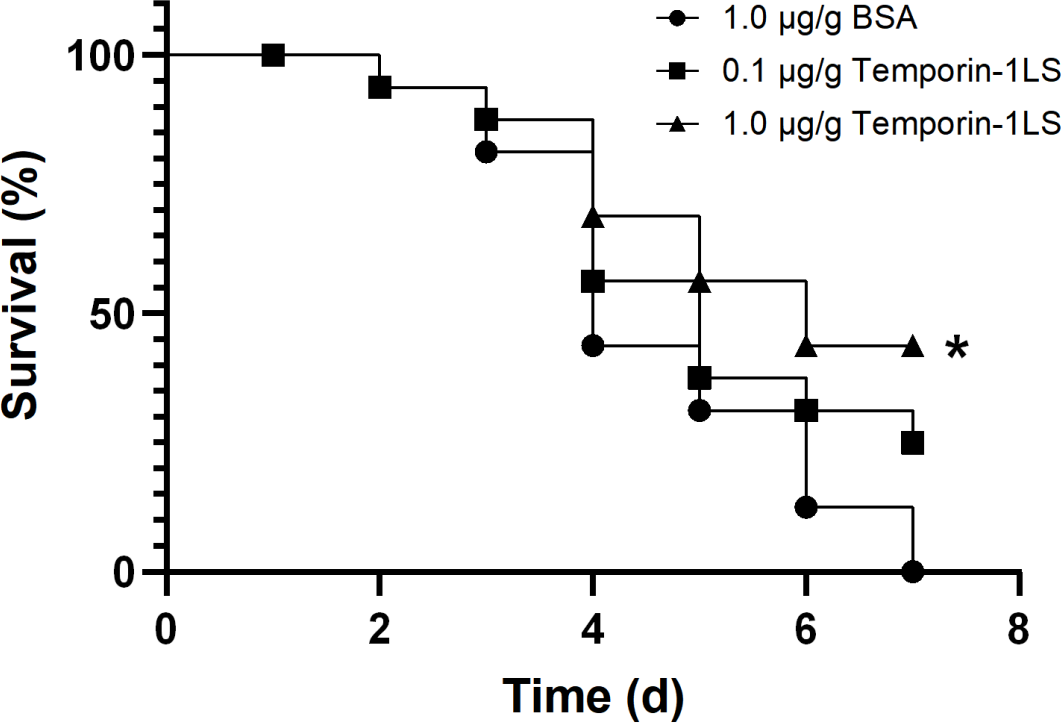
Effects of temporin-1LS on survival following *Aeromonas hydrophila* challenge. Frogs were intraperitoneally (i.p.) administered temporin-1LS (0.1 or 1.0 μg/g) at 30 min post-infection with *A. hydrophila*. Animals were examined for clinical signs and mortality every 12 h for 7 d (n = 16 per group). Survival curves were analyzed using the Kaplan–Meier method and compared by the log-rank (Mantel–Cox) test. *P < 0.05.

To confirm the effect of Temporin-1LS on the survival rate of *A. hydrophila*-infected dark-spotted frogs, the frogs were treated with Temporin-1LS following *A. hydrophila* infection. The results indicated that treatment with Temporin-1LS led to significant differences in survival rates among the experimental groups. By day 7, all frogs treated with BSA had died. The survival rate for frogs treated with 0.1 μg/g of Temporin-1LS was 25.00% on day 7. In contrast, the survival rate for frogs treated with 1.0 μg/g of Temporin-1LS was 43.75% on the same day (**Figure 6**).

### Effect of temporin-1LS on macrophage viability and chemotaxis

**Figure 7:**
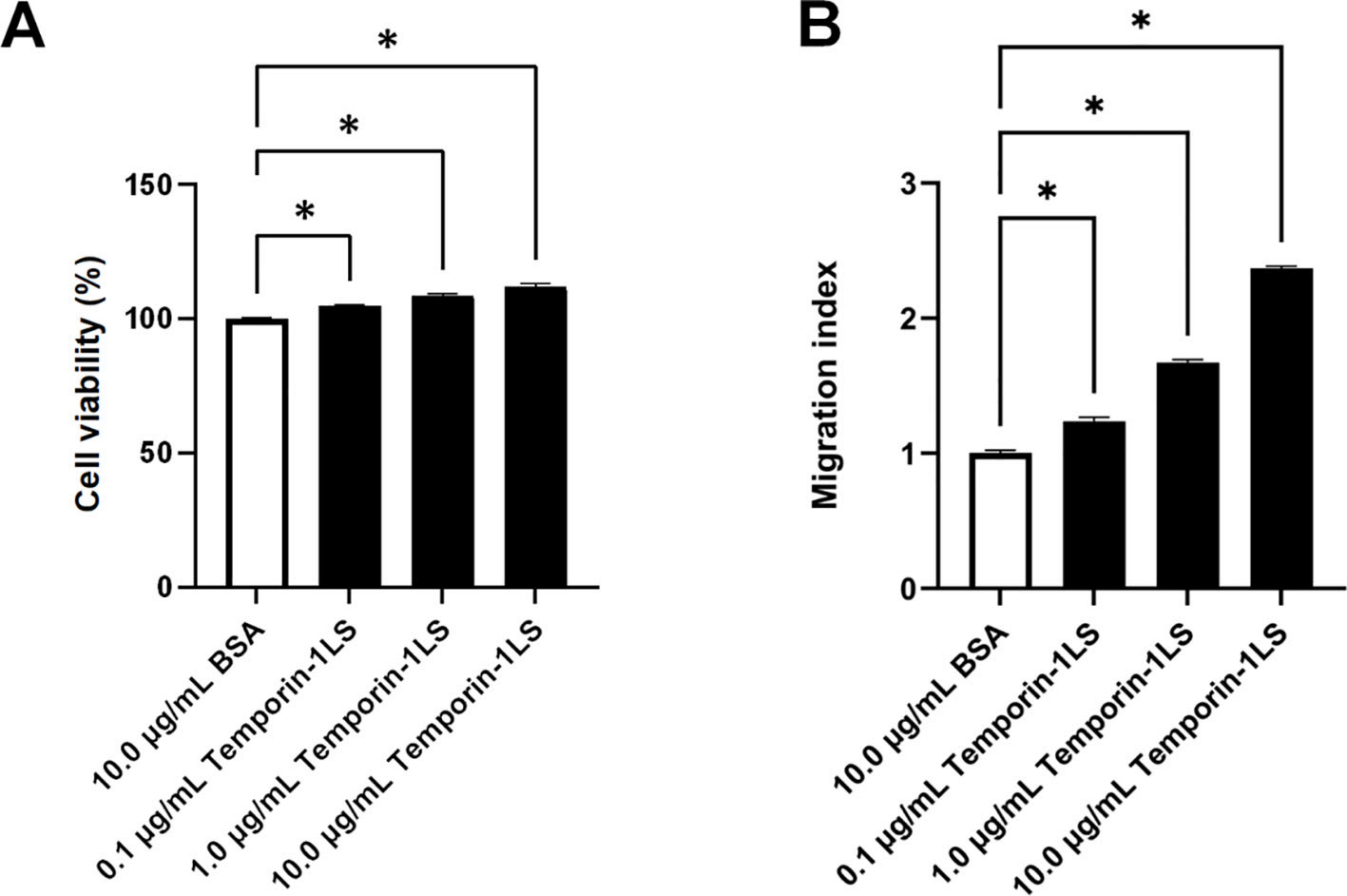
Effects of temporin-1LS on macrophage viability and chemotaxis. **(A)** Macrophage viability after 24 h exposure to temporin-1LS (0.1, 1.0, and 10.0 μg/mL), determined by the CCK-8 assay. **(B)** Macrophage chemotaxis toward temporin-1LS measured using Transwell chambers after 24 h. BSA served as the negative control in both assays. Data are presented as mean ± SEM from four (viability) or three (chemotaxis) independent experiments. Differences relative to the BSA control were analyzed by one-way ANOVA (*P < 0.05).

The CCK-8 assay results indicated that temporin-1LS showed no obvious cytotoxicity toward macrophages under the tested conditions. In addition, temporin-1LS increased the CCK-8 signal at concentrations of 0.1 μg/mL and above, suggesting enhanced cellular metabolic activity or viable cell-associated signal (**Figure 7A**).

Chemotactic potential was quantified using Transwell chambers with 24-hour migration assays. Temporin-1LS exhibited dose-responsive chemoattractant effects on macrophages across three log-scale concentrations (0.1–10.0 μg/mL), demonstrating 1.2-, 1.7-, and 2.4-fold increases in migrated cells compared to BSA controls (**Figure 7B**).

### Modulation of macrophage phagocytosis by temporin-1LS

**Figure 8:**
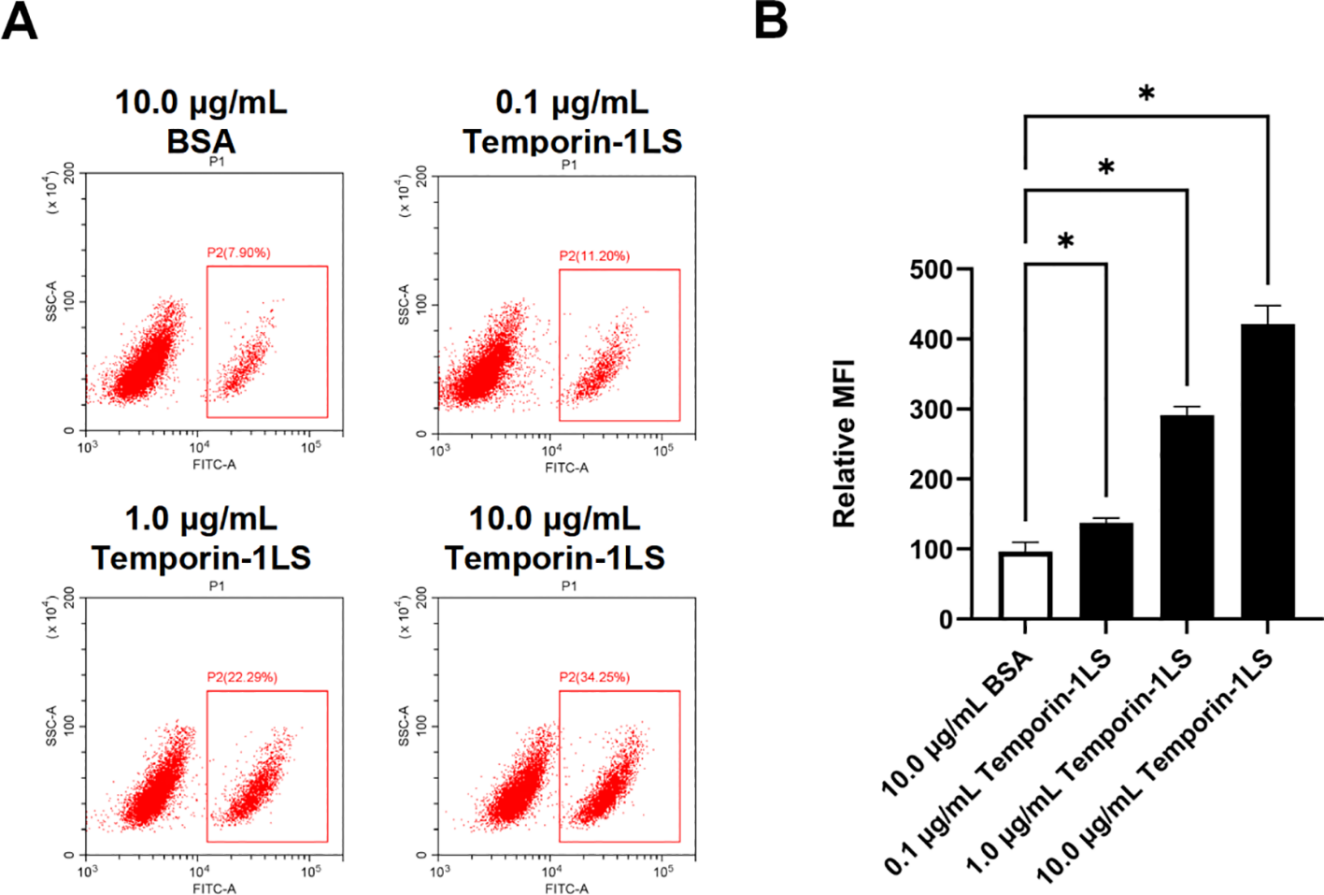
Temporin-1LS enhances macrophage phagocytosis. Phagocytic capacity was evaluated by quantifying FITC–dextran internalization, and the resulting MFI, mean fluorescence intensity was normalized to the BSA group (set to 100) and presented as relative change. Values are shown as mean ± SEM from three independent experiments. Group differences were assessed by one-way ANOVA, with *P < 0.05 considered significant.

The phagocytic activity of macrophages was systematically evaluated using flow cytometric analysis. Temporin-1LS potentiated FITC-dextran internalization in a concentration-dependent manner, demonstrating maximal enhancement at 10.0 μg/mL. This optimal concentration induced a 4.4-fold increase in phagocytic capacity relative to BSA-treated controls (**Figure 8**).

### Effect of temporin-1LS on macrophage respiratory burst

**Figure 9:**
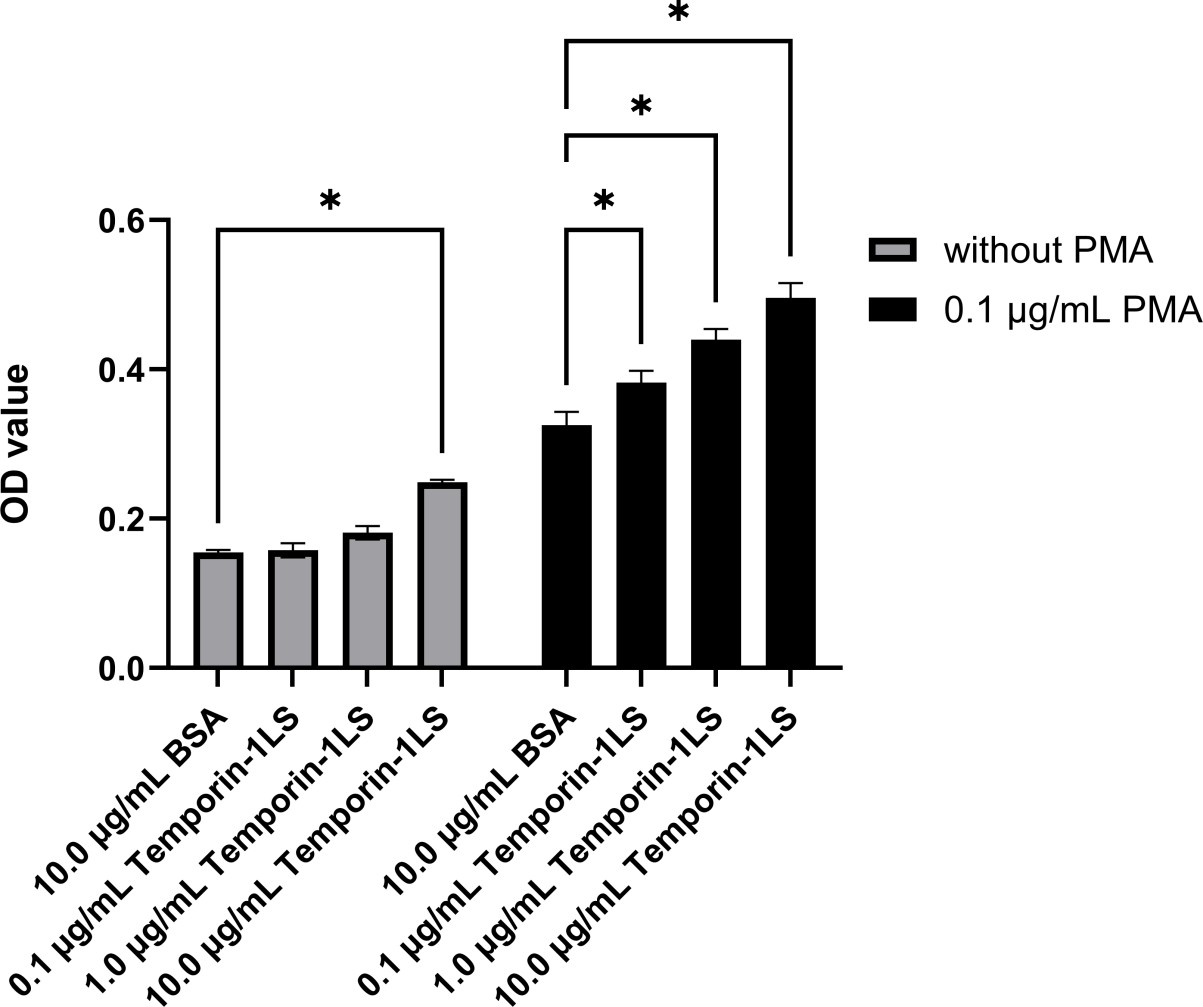
The impact of temporin-1LS on the respiratory burst of macrophages. Respiratory burst was quantified by measuring optical density at 620 nm (nitroblue tetrazolium chloride reduction assay). Data are expressed as mean ± SEM of three independent biological replicates. Statistical significance (*P < 0.05) was determined using one-way ANOVA.

Temporin-1LS induced a concentration-dependent enhancement of respiratory burst activity in macrophages. At 10.0 μg/mL, peptide treatment elevated reactive oxygen species production to 1.6-fold of BSA-treated controls. Synergistic amplification occurred upon co-stimulation with phorbol PMA, where 10.0 μg/mL temporin-1LS produced a 1.5-fold increase in respiratory burst compared to PMA+BSA co-treatment. The magnitude of this stimulatory effect exhibited clear dose-dependency across tested concentrations (**Figure 9**).

## Discussion

The identification and functional characterization of temporin-1LS from the dark-spotted frog provides compelling evidence for a sophisticated dual-defense strategy in amphibian host defense, which synergistically employs direct antimicrobial action and potent immunomodulation to combat infection.

Our findings confirm that temporin-1LS possesses the hallmark structural features of the temporin family—a short, amphipathic α-helix and a precursor-associated C-terminal Gly-Lys extension—which are characteristic of frog skin temporins (29, 34). In temporin peptides, the amphipathic α-helical conformation is closely related to membrane adsorption and perturbation, whereas the C-terminal Gly-Lys motif serves as an amidation signal for formation of the mature C-terminally amidated peptide (29). C-terminal amidation has been reported to enhance membrane interaction and, in some amphibian AMPs, to improve α-helical stabilization and antimicrobial efficacy (35, 36). However, these canonical temporin-like features do not necessarily confer broad-spectrum activity, and temporins are often characterized by mild cationicity and preferential activity against selected susceptible bacteria, especially Gram-positive species (34, 37). In this study, the calculated *p*I of temporin-1LS (5.5) suggests relatively limited overall cationicity, which may reduce electrostatic interactions with some bacterial envelopes and help explain its selective antibacterial profile. Thus, its spectrum is notably selective, as it showed no measurable *in vitro* growth-inhibitory activity against *A. hydrophila*. This selectivity is common among AMPs and may reflect differences in membrane composition among bacterial species (38) and in the balance among charge, hydrophobicity, helicity, and amphipathicity that governs AMP activity (39). The most striking evidence for a dual-defense mechanism came from our *in vivo* survival assay. Despite this lack of direct *in vitro* antibacterial activity, administration of temporin-1LS significantly protected frogs from a lethal *A. hydrophila* challenge. This important finding indicates that the peptide’s protective role in a live organism extends beyond, and can operate independently of, its direct *in vitro* antibacterial growth-inhibitory activity. It suggests that temporin-1LS confers survival advantage by mobilizing the host’s own immune system.

This hypothesis is supported by our comprehensive suite of immunomodulatory assays. We demonstrated that temporin-1LS is a activator of key macrophage effector functions. It acted as a potent chemoattractant, recruiting macrophages to the site of infection. Once activated, these macrophages exhibited a dramatically enhanced capacity to engulf pathogens, as shown by the 4.4-fold increase in phagocytosis. Furthermore, temporin-1LS primed the macrophages for a stronger respiratory burst, a crucial mechanism for intracellular pathogen destruction. The observed dose-dependency of these effects underscores their biological relevance. In addition, the increased CCK-8 signal induced by temporin-1LS may also be immunologically meaningful, as it suggests enhanced macrophage metabolic activity and cellular responsiveness under the tested conditions, which could help support innate immune surveillance, pathogen clearance, and immunoregulatory functions (40, 41). This coordinated enhancement of chemotaxis, phagocytosis, and respiratory burst represents a potent mechanism to amplify the innate immune response, effectively compensating for the peptide’s lack of direct activity against certain pathogens like *A. hydrophila*. This immunocentric defense strategy may help explain the improved survival in infected frogs.

The tissue-specific expression profile of temporin-1LS further illuminates its strategic role in host defense. Its constitutive high expression in the skin positions it as a critical first-line defender at the primary environmental interface (3). More remarkably, the massive induction in the gut (47.4-fold) upon systemic bacterial challenge suggests a pivotal and inducible role in mucosal immunity, a phenomenon reminiscent of mammalian defensin dynamics but less documented in amphibians. This pattern indicates a sophisticated defense system that maintains ready-to-deploy weapons at barrier sites while retaining the capacity to rapidly reinforce internal mucosal surfaces during systemic infection.

This study identifies temporin-1LS as a temporin-family dual-functional host defense peptide from the dark-spotted frog. Temporin-1LS displays selective antibacterial activity against a subset of tested bacteria (lowest MIC = 3.125 μg/mL) and may induce membrane damage in *S. warneri* at higher concentrations, as suggested by increased LDH release at 50–100 μg/mL relative to the BSA control. More importantly, temporin-1LS demonstrates significant immunomodulatory capabilities by markedly enhancing macrophage functions, including chemotaxis, phagocytosis, and respiratory burst. Crucially, *in vivo* experiments confirmed that the peptide significantly improved the survival of frogs infected with *A. hydrophila*—a pathogen it cannot kill directly—by activating the host’s immune system. This dual-defense mechanism, combining direct antibacterial activity with immunomodulation, positions temporin-1LS as a promising lead compound for developing novel anti-infective therapeutics against drug-resistant bacterial infections. However, the current safety evaluation remains preliminary, as it was based mainly on the hemolysis assay and the macrophage CCK-8 assay under the tested conditions. Additional toxicity assessments in other cell types and *in vivo* models will be necessary for a more comprehensive evaluation of its therapeutic and application potential.

## Data Availability

The datasets presented in this study can be found in online repositories. The names of the repository/repositories and accession number(s) can be found below: https://www.ncbi.nlm.nih.gov/genbank/, PQ826831.
